# Subjective and objective measures of sleep-related function from the Cardiovascular Endpoints For Obstructive Sleep Apnea with Twelfth Cranial Nerve Stimulation (CARDIOSA-12) trial: Sleep-related functional outcomes in CARDIOSA-12

**DOI:** 10.1007/s44470-026-00067-x

**Published:** 2026-04-07

**Authors:** Akshay Tangutur, Phoebe K. Yu, Mathias Basner, Brendan T. Keenan, Raj C. Dedhia

**Affiliations:** 1 Department of Otorhinolaryngology, University of Pennsylvania, 301 South 8th Street, Philadelphia, PA 16106 USA; 2 Unit for Experimental Psychiatry, Division of Sleep and Chronobiology, Department of Psychiatry, University of Pennsylvania, Philadelphia, PA USA; 3 Division of Sleep Medicine, Department of Medicine, University of Pennsylvania, Philadelphia, PA USA

**Keywords:** Obstructive sleep apnea, Hypoglossal nerve stimulation, Patient-reported outcomes, Cognitive, Randomized controlled trial

## Abstract

**Purpose:**

The effect of therapeutic versus partially therapeutic levels of hypoglossal nerve stimulation (HGNS) therapy on cognitive measures and patient-reported outcomes (PROs) was evaluated in patients with moderate-severe obstructive sleep apnea (OSA).

**Methods:**

In a 10-week, double-blind, randomized crossover therapy trial, subjects optimized on HGNS therapy underwent 4 weeks each of active and control (partially therapeutic) HGNS therapy. Cognitive measures (psychomotor vigilance test [PVT], digit symbol substitution test [DSST]) and sleep-related PROs were assessed after each 4-week treatment period.

**Results:**

Sixty subjects were randomized and completed the primary study protocol, including PROs. A subset of randomized subjects (*n* = 43) underwent cognitive testing. This subset was, on average, older (65.7 ± 10.2 years), overweight/obese (body mass index [BMI] 29.7 ± 4.6 kg/m^2^), and had severe OSA at baseline (apnea–hypopnea index [AHI] 34.2 ± 15.0 events/h). There were no differences in cognitive performance measures between active and control (partially therapeutic) HGNS conditions in all subjects; in the per-protocol analysis, however, improvement in DSST reaction time with active HGNS was demonstrated (mean [95% CI] change on active therapy = − 153.6 [− 285.3, − 22.0] ms; *p* = 0.025). Significant improvements in all PROs (Epworth Sleepiness Scale, Insomnia Severity Index, Functional Outcomes of Sleep Questionnaire, and Snoring Visual Analog Scale) were observed in the active HGNS condition.

**Conclusions:**

In this secondary analysis of the CARDIOSA-12 randomized crossover trial of subjects using HGNS, subjective PROs, but not objective cognitive measures, were improved with active HGNS compared to control (partially therapeutic) HGNS therapy. These findings warrant additional investigation examining the relationship between subjective and objective neurocognitive outcomes in OSA.

**Brief summary:**

The effect of hypoglossal nerve stimulation (HGNS), a novel and promising therapy for obstructive sleep apnea (OSA), on neurocognitive deficits remains underexplored. This study evaluated whether HGNS therapy improves cognitive performance using the psychomotor vigilance test (PVT) and digit symbol substitution test (DSST), along with patient-reported outcomes (PROs) related to sleepiness, snoring, insomnia, and sleep-related function. In the modified intention-to-treat analysis, no significant differences in PVT or DSST outcomes were observed between active and control (partially therapeutic) HGNS therapy; however, in a subset of participants with ≥ 50% reduction in AHI with active HGNS, improvement in DSST reaction time was noted. All PROs significantly improved with active HGNS, suggesting benefits in subjective measures without substantial changes in objective cognitive measures.

**Supplementary Information:**

The online version contains supplementary material available at 10.1007/s44470-026-00067-x.

## Introduction

Obstructive sleep apnea (OSA) is a sleep-related breathing disorder characterized by repetitive collapse of the upper airway resulting in frequent arousals and intermittent hypoxemia [1]. Untreated OSA has broad public health and economic consequences, including lost productivity and motor vehicle accidents, with an economic burden amounting to nearly $150 billion annually [2]. OSA is also associated with adverse patient-reported outcomes (PROs) and quality of life, including increased daytime sleepiness, reduced functional status, and worsened mood [3–5]. These symptoms may often interfere with occupational performance, interpersonal relationships, and daily activities. Untreated OSA also adversely affects cognition, with literature suggesting impairment in the domains of memory, attention, and/or executive function in untreated patients [6], with consequences of developing affective disorders and decreased activities of daily life (ADLs) [7–9]. Despite these consequences, the effect of OSA treatment on neurocognitive outcomes is not well understood.

Positive airway pressure (PAP) therapy is the first-line treatment for adults with OSA, and studies assessing the neurocognitive outcomes after PAP therapy have had mixed results [6, 10, 11]. The Apnea Positive Pressure Long-term Efficacy Study (APPLES), a sham-controlled, double-blind, randomized controlled trial, found that PAP therapy only improved executive/frontal lobe function at 2 months, with no significant differences reported for attention/psychomotor and learning/memory function at any time point [12]. A major limitation of APPLES was that participants were largely cognitively intact at baseline, potentially limiting the ability to detect meaningful improvements. Poor adherence rates with PAP also present a major challenge in this area of research and clinical care [13]. The literature surrounding oral appliance (OA) therapy, a titratable treatment to advance the mandible and open the airway, is also limited and with mixed results [14, 15]. Another alternative to PAP therapy is hypoglossal nerve stimulation (HGNS), which is well-tolerated with high adherence rates and becoming increasingly accessible [16, 17]. The effects of HGNS on neurocognitive outcomes has not been well-studied.

The objective of this secondary analysis is to evaluate the effects of HGNS therapy on objective measures of cognition and subjective patient-reported outcomes. Herein, we describe neurocognitive results from the Cardiovascular Endpoints for Obstructive Sleep Apnea With Twelfth Cranial Nerve Stimulation (CARDIOSA-12) randomized crossover trial of HGNS therapy [18]. Our primary hypothesis was that there would be a significant improvement in performance measures on cognitive assessments with active HGNS therapy compared to the control (partially therapeutic) condition; secondary hypotheses included expected improvements in sleep-related patient-reported outcomes with active HGNS therapy.

## Methods

### Trial design

This study utilizes data from the CARDIOSA-12 randomized crossover clinical trial [19]. In short, this 10-week trial consisted of two 4-week treatment periods, active vs. control (partially therapeutic) HGNS, each preceded by a 1-week washout. Subjects already implanted and adherent to their Inspire® HGNS device (Inspire Medical Systems, Inc.; Minnesota, USA) were enrolled from three academic medical centers. All participants and study team members except for the primary research coordinator were blinded to treatment order in an attempt to mitigate biased responses and assessment of subjective outcomes. The trial was approved by the University of Pennsylvania Institutional Review Board (IRB) and written informed consent was obtained from all participants.

The control (partially therapeutic) condition was defined as the average voltage at which bulk tongue motion without obvious protrusion was first observed following incremental titration (0.1–0.2 V) under mouth-open, nasal-breathing conditions. The same electrode configuration used for the therapeutic HGNS condition was maintained. For additional details regarding the trial’s subject eligibility criteria, randomization scheme and blinding procedures, and power/sample size calculations, see previous publications [18, 19]. In the CARDIOSA-12 randomized crossover trial, the control HGNS condition resulted in modest physiologic improvements in AHI and is therefore better interpreted as partially therapeutic HGNS when interpreting the results of this present study.

### Protocol changes

The addition of cognitive measures was a protocol amendment associated with the trial’s transfer between institutions with the principal investigator’s institutional transfer in 2019. Subjects enrolled following the trial’s transfer (*N* = 43) underwent cognitive assessments, while all randomized subjects included in the final analysis set (*N* = 60) were administered questionnaires to measure sleep-related patient-reported outcomes.

### Cognitive assessments

After each 4-week treatment period, participants were administered computerized versions of the psychomotor vigilance test (PVT) and the digit symbol substitution test (DSST) from a comprehensive neuropsychological test battery, *Cognition *[20].

The PVT is a validated measure of sustained attention based on reaction time to visual stimuli occurring at random intervals [21]. Here, we used a validated brief version of the PVT (PVT-B) with a 3-min test duration, random 2–5 inter-stimulus intervals, and a 355-ms lapse threshold [22]. The DSST is a computerized adaptation of a Wechsler Adult Intelligence Scale (WAIS-III) assessment that assesses visual search, spatial memory, paired associate learning, and sensory-motor speed [21]. Participant performance concentrated on reaction time and accuracy outcomes. The primary reaction time outcome for the PVT was the mean of reciprocal reaction time (PVT Mean RRT; 1/ms), with a higher reciprocal value indicating better performance. The primary reaction time outcome for the DSST was mean reaction time (DSST mean RT; milliseconds), with a lower value indicating better performance. Secondary outcomes of accuracy included transformed lapses (defined as responses > 355 ms) and false starts (defined as premature responses prior to stimulus onset) on the PVT and the number of correct responses and % accuracy on DSST. While the PVT has negligible learning effects [23], data suggest learning effects on the DSST and, thus, data were corrected to account for any order effects [21]. A full description of the PVT, DSST, and *Cognition* test battery are provided by Basner et al. [20].

PVT and DSST were performed using *Cognition* software v3.0.9 (Pulsar Informatics, Philadelphia, PA) on a Dell Latitude 7290 laptop with an Intel® Core™ i7-8650U (1.90 GHz) processor with Windows 10 Enterprise. The laptop was calibrated for timing accuracy prior to administering *Cognition*. All participants completed their cognitive testing alone in a quiet clinic exam room without interruptions using the same laptop to standardize the testing environment across administrations.

### Patient-reported outcomes

Participants were administered sleep-related PROs at trial entry, as well as after each 4-week treatment period. By utilizing validated instruments, we assessed changes in measures of sleepiness (Epworth Sleepiness Scale; ESS), snoring (Snoring Visual Analog Scale; Snoring VAS), insomnia (Insomnia Severity Index; ISI), and sleep-related functional outcomes (Functional Outcomes of Sleep Questionnaire-10; FOSQ-10) between active and control (partially therapeutic) HGNS treatments. For full details regarding sleep-related instruments, please refer to Lam et al. [24].

### Statistical methods

As the CARDIOSA-12 randomized crossover trial was powered for cardiovascular endpoints, analyses of cognitive outcomes and PROs in this study represent secondary, exploratory analyses and were not based on a priori power calculations.

#### Primary, secondary, and exploratory outcomes

The primary outcomes of interest were mean RRT on PVT and mean RT on DSST. The other measures obtained from cognitive testing were considered exploratory. Secondary outcomes included mean responses in sleep-related PROs.

#### Statistical analysis

Continuous data are summarized using means and standard deviations and categorical data are summarized using frequencies and percentages. Given the randomized crossover design in which each participant is their own control, statistical analyses comparing control (partially therapeutic) vs. active HGNS therapy were performed using methods for paired data (i.e., paired *T*-tests for differences in continuous measures). In addition to analyses within the full sample, we used statistical interaction tests and stratified analyses to compare differences between HGNS therapy conditions in subgroups defined based on randomized therapy order (control first vs. active first), higher vs. lower mean RRT on PVT (based on a median split), and higher vs. lower mean RT on DSST (based on a median split). A Hochberg step-up procedure was utilized to control for multiple comparisons in primary and secondary outcome analyses [25, 26].

#### Analysis sets

**Fig. 1:**
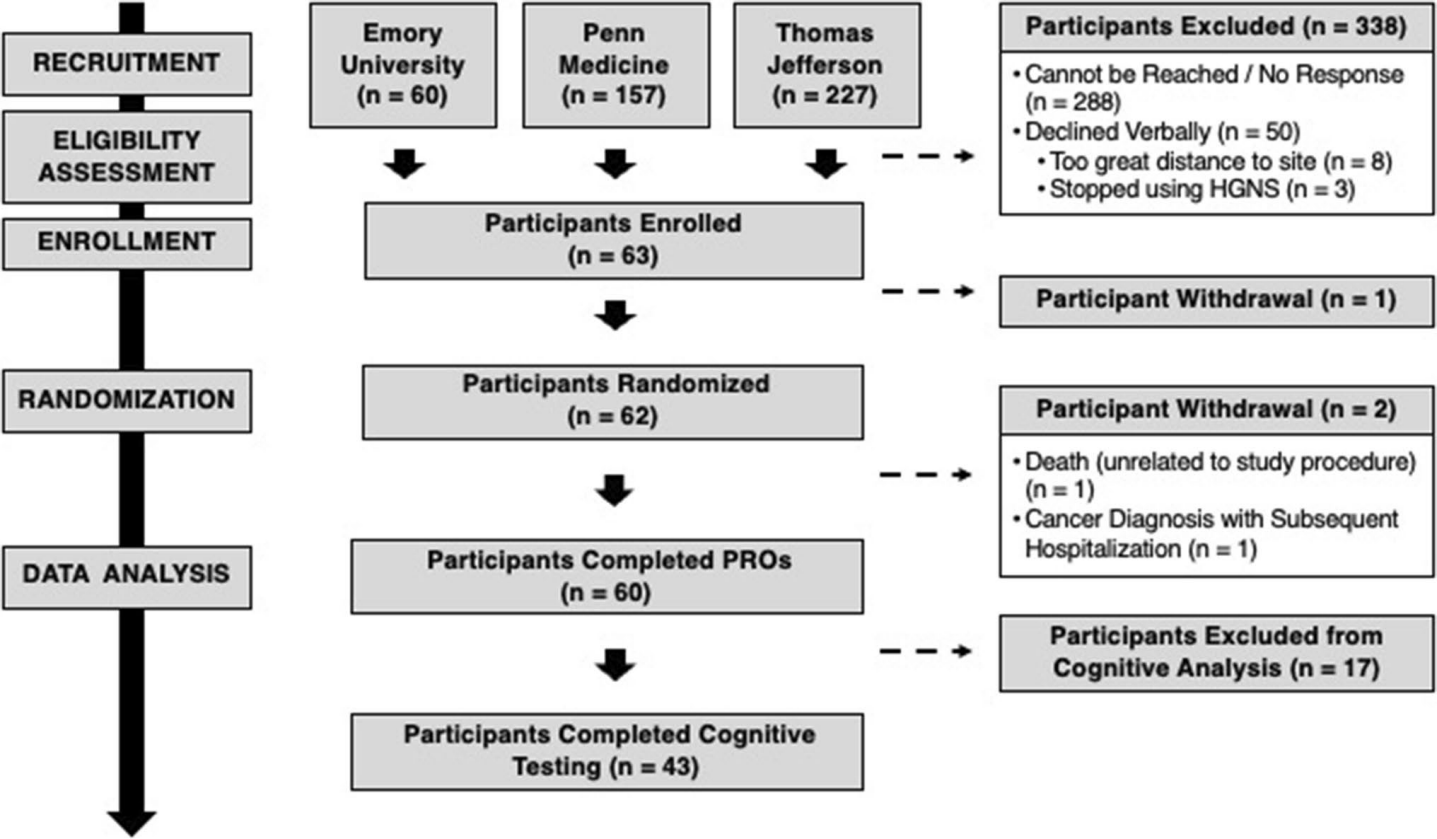
CONSORT diagram. HGNS indicates hypoglossal nerve stimulation; PROs indicate patient-reported outcomes

Primary analyses were performed in a *modified intent-to-treat* (mITT) analysis set, defined as all randomized participants who completed both arms of the study. This included a total of 60 participants for patient-reported outcomes analysis and 43 participants who underwent cognitive testing (Fig. 1). We also defined a *per-protocol* (PP) analysis set as individuals that exhibited a ≥ 50% improvement in AHI with active vs. control (partially therapeutic) HGNS therapy, informed by the surgical success criteria originally described by Sher et al., but applied in this study as a simplified and more liberal adaptation [27]. This included 20 participants for analyses of sleep-related PROs and 15 participants in analyses of cognitive outcomes.

## Results

### Analysis sample

The overall flow of participants is illustrated in a CONSORT diagram (Fig. 1). Notably, certain CONSORT elements, such as pre-specification of cognitive outcomes and outcome-specific sample size determination, are not applicable to this present study as cognitive assessments were introduced following trial initiation.

In this secondary analysis of the CARDIOSA-12 randomized crossover therapy trial, 63 subjects were enrolled, 62 were randomized, and 60 completed the trial. Among the 60 subjects who completed the trial, only subjects enrolled after the trial’s institutional transfer (*N* = 43) underwent cognitive testing (Fig. 1). Clinical characteristics of the full mITT analysis set are presented in Table 1; similar data for the full per-protocol analysis set are presented in Table S1. Randomized participants who completed cognitive testing were, on average, elderly (65.7 ± 10.2 years), borderline obese (BMI 29.7 ± 4.6 kg/m^2^), and predominantly male (70%) and White (93%). Participants had severe OSA (AHI 34.2 ± 15.0 events/h) prior to initiating HGNS therapy. Complete descriptive statistics for the subset of the mITT (Table S2) and PP (Table S3) analysis sets that completed cognitive testing are provided in the Supplemental Material; in general, this cohort had similar characteristics to the full sample.

**Table 1:** Demographics of the modified intent-to-treat analysis set

Characteristic	Mean (± SD) or %			p*
	All	Control first	Active first	
Total *N*	60	31	29	–
Gender, %				
Male	63.3%	64.5%	62.1%	0.844
Female	36.7%	35.5%	37.9%	
Age, years	67.3 ± 9.9	67.1 ± 11.8	67.6 ± 7.5	0.848
< 70	56.7%	51.6%	62.1%	0.414
≥ 70	43.3%	48.4%	37.9%	
Race, %				
White	91.7%	93.6%	89.7%	0.164
Black	5.0%	0.0%	10.3%	
Hispanic	1.7%	3.2%	0.0%	
Asian	1.7%	3.2%	0.0%	
BMI, kg/m^2^	28.7 ± 4.6	27.9 ± 4.2	29.6 ± 4.9	0.157
< 30	60.0%	71.0%	48.3%	0.073
≥ 30	40.0%	29.0%	51.7%	
Diabetes, %	24.6%	17.9%	31.0%	0.248
Hypertension, %	57.9%	60.7%	55.2%	0.672
Hypertension meds, %	59.7%	60.7%	58.6%	0.872
Smoking status, %				
Current	10.0%	12.9%	6.9%	0.037
Never	45.0%	58.1%	31.0%	
Former	45.0%	29.0%	62.1%	
Pre-implant AHI, events/hour	33.1 ± 14.9	31.9 ± 14.9	34.4 ± 15.0	0.509
HGNS configuration				
±/+	75.0%	74.2%	75.9%	0.217
-/-/-	5.0%	0.0%	10.3%	
-/○/-	8.3%	9.7%	6.9%	
○/-/○	11.7%	16.1%	6.9%	

^*^p-value from *T*-test or chi-squared test comparing participants randomized to control condition first or active therapy first

### AHI response and treatment-related metrics

The preoperative baseline AHI was 33.1 ± 14.9 events/h. AHI decreased to an average of 23.0 ± 15.6 events/h following 4 weeks of control (partially therapeutic) HGNS therapy (*p* = 0.0001) and 18.1 ± 14.8 events/h following 4 weeks of active therapy (*p* < 0.0001). The AHI was − 4.9 [− 8.8, − 1.0] events/h lower with active HGNS therapy versus control (partially therapeutic) condition (*p* = 0.014). Participants demonstrated a 1.4-h (95% CI [1.04, 1.84] h) greater adherence to control (partially therapeutic) HGNS therapy (mean [SD], 8.19 [1.22] hours/night) compared to active HGNS therapy (mean [SD], 6.80 [1.74] h/night). ODI was an average of 22.0 ± 15.3 events/h following 4 weeks of control (partially therapeutic) HGNS therapy and 16.7 ± 14.3 events/h following 4 weeks of active therapy. Finally, %TST < 90% was an average of 6.79 ± 10.3% following 4 weeks of control (partially therapeutic) HGNS therapy and 5.25 ± 10.7% following 4 weeks of active therapy.

### Results in the modified intent-to-treat analysis set

Results of primary analyses comparing control (partially therapeutic) and active HGNS treatment within the mITT analysis set are shown in Table 2 and discussed below with respect to the co-primary (PVT, DSST), secondary (sleep-related PROs), and exploratory cognitive outcomes.

**Table 2:** Comparisons of outcomes during control condition and active therapy among mITT analysis set

Clinical measures	Control		Active		Difference^*^		***p***^†^
	***N***	Mean ± SD	***N***	Mean ± SD	***N***	Mean (95% CI)	
Cognitive measures							
**PVT (mean RRT)**^§^	**43**	**3.97 ± 0.51**	**43**	**4.01 ± 0.47**	**43**	**0.05 (−0.03, 0.13)**	**0.239**
PVT (lapses)^‡^	43	4.60 ± 4.79	43	4.07 ± 3.89	43	− 0.53 (− 1.57, 0.50)	0.302
PVT (false starts)^‡^	43	1.64 ± 2.16	43	2.07 ± 2.71	43	0.44 (− 0.02, 0.89)	0.060
**DSST (mean RT)**^‡^	**43**	**1893.6 ± 428.1**	**43**	**1846.6 ± 355.5**	**43**	− **46.9 (**− **113.1, 19.2)**	**0.160**
DSST (# correct)^§^	43	42.77 ± 8.59	43	43.05 ± 8.32	43	0.28 (− 1.33, 1.89)	0.728
DSST (% accuracy)^§^	43	0.98 ± 0.03	43	0.97 ± 0.05	43	− 0.01 (− 0.03, 0.01)	0.356
**ESS total**^‡^	**59**	**7.22 ± 4.86**	**60**	**5.50 ± 3.67**	**59**	− **1.75 (**− **2.62,** − **0.88)**	**0.0002**
**Snoring VAS**^‡^	**57**	**37.26 ± 29.69**	**57**	**20.95 ± 25.25**	**56**	− **15.50 (**− **23.53,** − **7.47)**	**0.0003**
**ISI total**^‡^	**59**	**10.93 ± 5.31**	**60**	**8.77 ± 5.71**	**59**	− **2.37 (**− **3.76,** − **0.99)**	**0.0011**
**FOSQ total**^§^	**59**	**16.93 ± 2.36**	**60**	**17.69 ± 2.42**	**59**	**0.88 (0.40, 1.35)**	**0.0005**

^*^Difference calculated as active minus control condition

^†^*p*-value from paired T-test evaluating significance of within-subject changes on active therapy vs. control condition

^‡^Decreases with active therapy considered positive treatment benefit

^§^Increases with active therapy considered positive treatment benefit; primary and co-secondary measures shown in bold

#### PVT and DSST outcomes

There were no statistically significant differences between active and control (partially therapeutic) HGNS therapy in the primary outcome measures; however, observed changes in the mean RRT on the PVT (mean [95% CI] change on active therapy = 0.05 [− 0.03, 0.13] 1/ms; *p* = 0.239) and mean RT on the DSST (mean [95% CI] change on active therapy = − 46.9 [− 113.1, 19.2] ms; *p* = 0.160) trended in the hypothesized direction. There were also no differences observed between active and control (partially therapeutic) HGNS therapy in exploratory measures of accuracy on either PVT or DSST (see Table 2).

#### Patient-reported outcomes

Significant differences in the hypothesized direction were observed between active and control (partially therapeutic) HGNS therapy for all secondary patient-reported outcomes (*p* ≤ 0.001), with mean (95% CI) changes on active therapy of − 1.75 (− 2.62, − 0.88) for the ESS, − 15.50 (− 23.53, − 7.47) for the Snoring VAS, − 2.37 (− 3.76, − 0.99) for the ISI, and 0.88 (0.40, 1.35) for the FOSQ (see Table 2).

### Blinding adequacy

Among 58 individuals who completed the blinding adequacy assessment at trial completion, 51 (87.9%) correctly guessed their treatment order, which is significantly greater than the expected 50% (*p* < 0.0001). When blinding adequacy was assessed 2 days following the first treatment arm, 41 of 55 responders (74.5%) correctly guessed their treatment, again significantly greater than the expected 50% (*p* = 0.0001). This suggests that, despite blinding to randomization order, subjects may have been able to determine whether they were receiving active versus control (partially therapeutic) therapy.

### Per-protocol analyses

**Fig. 2:**
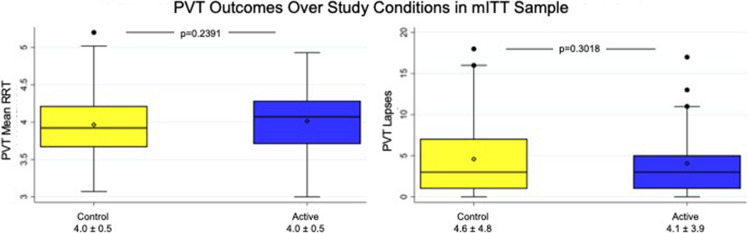
Comparison of psychomotor vigilance (PVT) outcomes in modified intention-to-treat (mITT) analysis set. PVT indicates psychomotor vigilance test; RRT indicates reciprocal reaction time

**Fig. 3:**
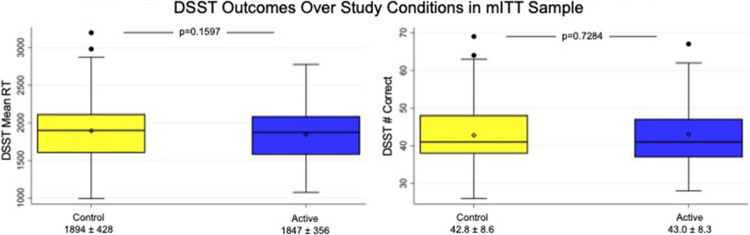
Comparison of digit symbol substitution test (DSST) outcomes in modified intention-to-treat (mITT) analysis set. DSST indicates digit symbol substitution test; RT indicates reaction time

We repeated analyses within a per-protocol analysis set defined by an AHI reduction between control (partially therapeutic) and active HGNS treatment of at least a difference of 50% (Table 3). In this analysis, a statistically significant difference was observed between active and control (partially therapeutic) HGNS therapy in mean RT on the DSST (mean [95% CI] change on active therapy = − 153.6 [− 285.3, − 22.0] ms; *p* = 0.025). No significant difference was seen for mean RRT on the PVT or for exploratory measures on the PVT and DSST (Figs. 2 and 3). Regarding sleep-related PROs, significant differences were again observed between active and control (partially therapeutic) HGNS therapy, with mean (95% CI) changes on active therapy of − 22.32 (− 35.99, − 8.64) for the Snoring VAS (*p* = 0.003), − 3.25 (− 5.44, − 1.06) for the ISI (*p* = 0.006), and 0.84 (0.19, 1.50) for the FOSQ (*p* = 0.015); no statistically significant difference was seen in the ESS.

**Table 3:** Comparisons of outcomes during control condition and active therapy among PP analysis set

Clinical measures	Control		Active		Difference^*^		***p***^†^
	***N***	mean ± SD	N	mean ± SD	N	mean (95% CI)	
Cognitive measures							
**PVT (mean RRT)**^**§**^	**15**	**3.85 ± 0.56**	**15**	**3.89 ± 0.44**	**15**	**0.04 (−0.09, 0.18)**	**0.504**
PVT (lapses)^‡^	15	4.60 ± 4.76	15	3.93 ± 3.81	15	− 0.67 (− 2.02, 0.68)	0.308
PVT (false starts)^‡^	15	1.62 ± 1.67	15	1.66 ± 2.06	15	0.04 (− 0.42, 0.51)	0.844
**DSST (mean RT)**^**‡**^	**15**	**2055.0 ± 514.5**	**15**	**1901.4 ± 430.1**	**15**	− **153.6 (**− **285.3,** − **22.0)**	**0.025**
DSST (# correct)^§^	15	40.20 ± 8.15	15	42.40 ± 9.07	15	2.20 (− 0.76, 5.16)	0.133
DSST (% accuracy)^§^	15	0.99 ± 0.03	15	0.97 ± 0.03	15	− 0.02 (− 0.04, 0.01)	0.141
**ESS total**^**‡**^	**20**	**7.40 ± 5.41**	**20**	**6.25 ± 3.61**	**20**	− **1.15 (**− **2.81, 0.51)**	**0.163**
**Snoring VAS**^**‡**^	**20**	**38.55 ± 32.51**	**19**	**15.00 ± 20.54**	**19**	− **22.32 (**− **35.99,** − **8.64)**	**0.003**
**ISI total**^**‡**^	**20**	**10.85 ± 6.38**	**20**	**7.60 ± 5.24**	**20**	− **3.25 (**− **5.44,** − **1.06)**	**0.006**
**FOSQ total**^**§**^	**20**	**17.20 ± 2.31**	**20**	**18.04 ± 1.87**	**20**	**0.84 (0.19, 1.50)**	**0.015**

^*^Difference calculated as active minus control condition

^†^*p*-value from paired T-test evaluating significance of within-subject changes on active therapy vs. control condition

^‡^Decreases with active therapy considered positive treatment benefit

^§^Increases with active therapy considered positive treatment benefit; Primary and co-secondary measures shown in bold

### Subgroup analyses

In the mITT analysis set, we first explored whether the differences between active and control (partially therapeutic) HGNS therapy differed based on randomized order (see Table S4). There was some evidence that those randomized to control (partially therapeutic) condition first showed greater improvements with active therapy in the mean RT on DSST (mean [95% CI] difference = − 110.0 [− 209.3, − 10.7] ms) than those randomized to active first (19.1 [− 66.6, 104.8] ms; interaction *p* = 0.048). There was also evidence of better responses in those randomized to control (partially therapeutic) condition first for exploratory measures of PVT lapses (interaction *p* = 0.005) and DSST % accuracy (interaction *p* = 0.026) (Table S4). There were no differences in PRO responses based on randomized order.

We also compared effects of HGNS in subgroups defined by median splits of the control (partially therapeutic) therapy values of mean RRT on the PVT or mean RT on the DSST (Table S4). There was evidence that those with worse (slower) PVT mean RRT on control (partially therapeutic) HGNS therapy had greater improvements in mean RRT with active therapy (0.15 [0.02, 0.27] 1/ms) than those in the faster 50^th^ percentile (− 0.04 [− 0.14, 0.07] 1/ms) (interaction *p* = 0.026). Similarly, the mean [95% CI] change in DSST mean RT was better with active therapy for those in the slower 50^th^ percentile (− 111.3 [− 224.4, 1.9] ms; interaction *p* = 0.043) compared to those in the faster 50^th^ percentile (20.5 [− 42.9, 83.8] ms). There was also evidence of better responses in PVT lapses (interaction *p* = 0.047) and in snoring VAS (interaction *p* = 0.029) among those with *slower* PVT mean RRT, and in FOSQ among those with *slower* DSST response times (interaction *p* = 0.003).

## Discussion

In this secondary analysis of the CARDIOSA-12 randomized crossover therapy trial, objective measures of cognition were not significantly different between control (partially therapeutic) and active HGNS levels. All sleep-related PROs, however, demonstrated statistically significant improvement on active HGNS therapy. While a significant improvement in reaction time on DSST was found in a per-protocol analysis set, cognitive outcomes and PROs were secondary endpoints of a trial powered for cardiovascular outcomes, and analyses presented in this study should be interpreted as exploratory and may be underpowered.

Our findings related to cognitive outcomes and sleep-related PROs for HGNS must be considered in the context of well-established OSA therapies [6, 10, 11, 14, 15]. The impact of PAP, oral appliance (OA), and surgical interventions on *subjective* sleep-related symptoms has been extensively documented, with multiple studies demonstrating associations between treatment adherence and improvements in measures like the ESS, ISI, Snoring VAS, and FOSQ-10 [28–34]. However, the evidence regarding the effect of these therapies on *objective* cognitive outcomes remains inconsistent. Most of the literature has focused on PAP therapy, with relatively few studies examining performance-based measures such as the PVT or DSST. While prior studies compared effective OSA therapy to untreated disease, the present trial compares fully optimized HGNS to a partially therapeutic HGNS condition. This distinction therefore limits direct comparability to the following studies evaluating patients with untreated OSA. Bhat et al. reported PAP therapy improved performance on PVT mean RRT primarily in subjects with severe OSA (baseline mean RRT of 3.74 ± 0.6 1/ms vs. 3.81 ± 0.5 with PAP therapy; *p* = 0.07), though high dropout rates due to therapy non-adherence introduced potential selection bias [35]. Deering et al. similarly found a positive association between PAP adherence and PVT accuracy [36], while Richards et al. reported significant improvements in Wechsler Adult Intelligence Scale (WAIS) Digit Symbol subtest performance among subjects with both OSA and mild cognitive impairment receiving PAP therapy [37]. Studies evaluating OA therapy and surgical interventions have also suggested cognitive benefits. For instance, Gupta et al. demonstrated improved PVT performance following 6 months of OA therapy [38], and a multicenter trial of maxillomandibular advancement reported similar improvements [39]. In contrast, the literature on HGNS remains limited. The STAR trial, the landmark study of HGNS for OSA [40], reported improvements in ESS and FOSQ over 12 months, though commonly associated symptoms like insomnia and snoring were not assessed. Similarly, our study found improvements in ESS and FOSQ over a shorter 4-week period, though these outcomes are inherently vulnerable to response bias. To our knowledge, our study is the first to evaluate objective cognitive performance in patients with OSA treated with HGNS. Notably, Heiser et al. reported improved *alertness* in a small cohort of HGNS-treated patients [41]; however, their assessment involved the maintenance of wakefulness test (MWT), which, while relevant to cognitive function, is lengthy, impractical, and not a direct measure of cognition.

Our study highlights a recurring but incompletely understood phenomenon, which is patients with OSA often report “feeling better” following treatment despite a lack of measurable improvements in objective cognitive performance. This discordance between subjective improvement without consistent objective cognitive gains has been noted in prior studies evaluating PAP therapy [12], and there may be several potential explanations to help contextualize our findings. First, subjective PROs and performance-based objective cognitive tasks like PVT and DSST may assess distinct domains of cognitive function. Prior studies have found inconsistent correlations between the two in patients with OSA [12, 42], suggesting that perceived improvement does not necessarily equate with objective improvements. Our findings mirror this discordance seen in the literature, with participants reporting improved quality of life and symptom relief without corresponding improvements in reaction time or accuracy. Next, measured cognitive improvement may require a certain threshold of therapeutic efficacy. In our study, patients who achieved ≥ 50% reduction in AHI demonstrated significant improvements in reaction time compared to those with lesser AHI reductions as seen in our mITT and PP analyses. This may suggest that insufficient efficacy of HGNS treatment might blunt potential cognitive benefits. In a study conducted by Barnes et al. comparing PAP and OA therapy, patients receiving PAP therapy had the greatest AHI reduction and most improvement in Trail Making B (faster processing speed) and PVT lapses (fewer lapses) [43]. OA therapy, which resulted in a moderate AHI reduction, demonstrated similar but intermediate-level improvements in cognitive performance. Taken together with our results, this pattern suggests a possible association between the degree of AHI reduction and resultant cognitive improvement. Third, patients in our study experienced a modest and statistically significant reduction in AHI with control (partially therapeutic) HGNS, accompanied by small improvements in cognitive performance. A similar pattern was observed in the placebo arm of the study led by Barnes et al., where participants receiving a placebo tablet showed modest improvements in AHI, Trail Making B, and PVT outcomes. While control (partially therapeutic) HGNS is not directly equivalent to a placebo tablet, both represent “inactive” interventions that may influence outcomes through behavioral changes or placebo effects, suggesting even partially therapeutic interventions may have a partially therapeutic impact. Finally, exploratory analyses in our study revealed that participants in the slower 50th percentile for baseline PVT and DSST performance had greater improvements in reaction time compared to their faster-performing counterparts. While this may partly reflect regression to the mean, it does support the idea that baseline impairment relates to the magnitude of cognitive benefit. Subjects with minimal cognitive deficits at baseline may be less likely to demonstrate measurable cognitive change. While larger trials observe minimal improvements in cognitive performance [44], sometimes attributed to a ceiling effect due to high baseline cognitive functioning, few studies have directly compared treatment effects by baseline performance. This gap highlights the need for future studies to stratify cognitive outcomes by baseline performance to better understand which patients benefit the most from therapy.

The results of this study are limited and must be interpreted in the context of the design. Future studies evaluating HGNS therapy should be meticulous in selecting sham component criteria, as a limitation of our trial was the partially therapeutic effects of the control condition. For this reason, our analysis included a *per-protocol* analysis within a subset of subjects demonstrating at least a 50% reduction in AHI, but this sample had limited statistical power and an increased risk of a type II error. In addition, certain primary outcome measurements (PVT, DSST) were introduced later following the trial’s institutional transfer, and so not all subjects contributed to these analyses. The extent to which improvements observed on objective cognitive testing may translate into meaningful changes in daily functioning remains uncertain. Furthermore, the 4-week duration of each study arm may have been insufficient to capture the full extent of cognitive changes, although prior literature suggests that cognitive effects of untreated OSA can emerge within days to weeks [9]. Our blinding adequacy assessments showed that most subjects correctly guessed their treatment order at trial completion, which may limit the validity of the subjective assessments like PROs between control (partially therapeutic) vs. active HGNS. However, our blinding adequacy data appears to be consistent with randomized controlled trials involving surgical interventions, which are generally more difficult to successfully blind [45]. The magnitude of AHI reduction observed in this secondary analysis was also smaller than that reported in the overall CARDIOSA-12 cohort. This attenuation in response may reflect differences in participant characteristics among those undergoing cognitive testing. Additionally, HGNS efficacy was determined from single-night clinical sleep studies, consistent with standard of care, though this may not fully account for night-to-night variability in treatment response. Greater adherence during control (partially therapeutic) HGNS therapy may have also attenuated differences in both objective cognitive outcomes and PROs, therefore these findings warrant further investigation of adherence to HGNS in larger studies. While sleep-related PROs significantly improved on active HGNS (mean changes: − 1.75 for ESS, − 2.37 for ISI, and 0.88 for FOSQ), these changes were smaller than the minimal clinically important differences (MCIDs) of 2.65 for ESS, 6 for ISI, and 2.2 for the FOSQ [46–48]. The improvements observed in PROs may reflect a combination of modest physiologic benefit and expectancy effects. Finally, future studies incorporating comprehensive physiologic metrics, including pre- and postoperative hypoxic burden, are needed to better characterize treatment response to HGNS outcomes.

This study also bears several strengths. The “on/off” effect of HGNS permits a within-subject, therapeutic-controlled component via a randomized double-blind trial design, which is unique in surgical trials. Further, the use of a crossover design not only enabled our subjects to serve as their own controls to minimize confounding risk but also improved trial feasibility by reducing the required sample size. In addition to the rigorous trial design, the study team was able to ensure and quantify adherence to HGNS therapy during each intervention using the Inspire® Cloud adherence monitoring software. Finally, the cognitive assessments were corrected for practice effects that are commonly associated with serially administered objective assessments of cognition [21].

In conclusion, this randomized, double-blind, crossover HGNS study did not detect differences in objective cognitive outcomes between the control (partially therapeutic) and active conditions, while all subjective measures demonstrated improvement between conditions. This notable discrepancy has been identified in other sleep therapy trials and merits further consideration, namely “which one matters more?” Future research using long-term observational studies would serve as complementary data to the findings of this interventional study in answering this lingering, critical question in clinical sleep medicine.

## Supplementary Information

Below is the link to the electronic supplementary material.

ESM1 (JPG.28.9 KB)

ESM2 (JPG.30.5 KB)

ESM3 (DOCX.46.8 KB)

## Data Availability

Data collected for this study are deidentified, stored securely in a REDCap database, and are not publicly available due to privacy and institutional restrictions. Data may be made available from the corresponding author upon reasonable request and with institutional approval.

## Abbreviations

ADL: Activities of daily living: Basic self-care tasks that are essential for independent living, including washing, bathing, and toileting.
AHI: Apnea hypopnea index: A measure of sleep apnea severity, calculated as the number of apneas and hypopneas per hour of sleep.
BMI: Body mass index: A ratio of weight to height to better categorize individuals.
CI: Confidence interval: A statistical range to estimate uncertainty around a study result.
DSST: Digit symbol substitution test: A neuropsychological test that measures attention, processing speed, and executive function.
ESS: Epworth Sleepiness Scale: A validated questionnaire to assess daytime sleepiness based on the likelihood of sleeping in common scenarios.
FOSQ: Functional Outcomes of Sleep Questionnaire: A validated questionnaire to assess the impact of sleep disorders on daily functioning.
HGNS: Hypoglossal nerve stimulation: A surgical treatment for obstructive sleep apnea, involving a device that stimulates the hypoglossal nerve to maintain airway patency.
IRB: Institutional Review Board: A committee that reviews and approves research involving human subjects.
ISI: Insomnia Severity Index: A validated self-reported questionnaire to assess the severity of insomnia symptoms.
MCID: Minimal clinically important difference: The smallest change in a treatment outcome that would be identified as clinically meaningful.
mITT: Modified intention to treat: An analysis approach including all randomized participants with some modification.
OA: Oral appliance: A nonsurgical treatment for obstructive sleep apnea, involving a device worn in the mouth to reposition the tongue and/or mandible.
OSA: Obstructive sleep apnea: A sleep breathing disorder characterized by repetitive collapse of the upper airway.
PAP: Positive airway pressure: The gold standard treatment for obstructive sleep apnea, which involves delivering pressurized air to keep the airway patent during sleep.
PP: Per-protocol: An analysis approach that only includes participants who completed the trial according to the protocol.
PRO: Patient-reported outcomes: Health outcomes directly reported by the patient.
PVT: Psychomotor vigilance test: A neuropsychological reaction time test that measures attention and alertness.
RRT: Reciprocal reaction time: The inverse of the reaction time.
RT: Reaction time: The time elapsed between stimulus presentation and participant’s response.
VAS: Visual Analog Scale: A measurement tool to assess subjective characteristics.

## Declarations

### Trial registration

ClinicalTrials.gov.

### Trial URL

https://clinicaltrials.gov/ct2/show/NCT03359096
