# Supplementary figures and images for: Subjective and objective measures of sleep-related function from the Cardiovascular Endpoints For Obstructive Sleep Apnea with Twelfth Cranial Nerve Stimulation (CARDIOSA-12) trial: Sleep-related functional outcomes in CARDIOSA-12
Source: J Clin Sleep Med. 2026 Apr 7;22(1):45. doi: 10.1007/s44470-026-00067-x (PMC13057149; doi:10.1007/s44470-026-00067-x)

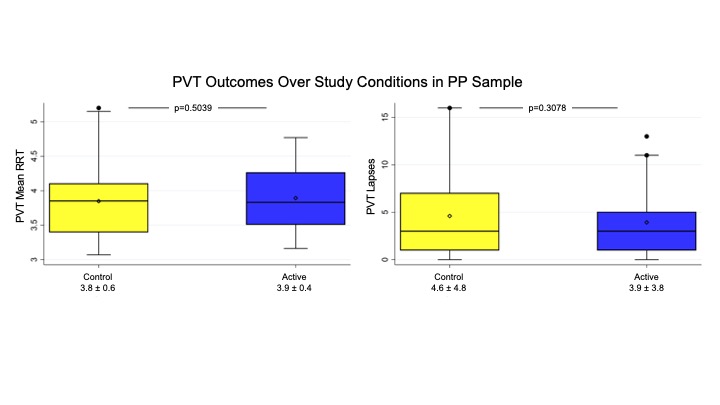

Supplement: Supplementary file 1 — (JPG.28.9 KB) [file 44470_2026_67_MOESM1_ESM.jpg]

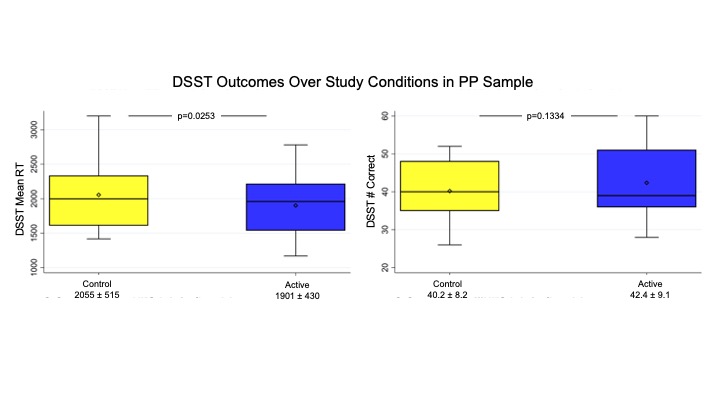

Supplement: Supplementary file 2 — (JPG.30.5 KB) [file 44470_2026_67_MOESM2_ESM.jpg]
