# Supplementary material for: Subjective and objective measures of sleep-related function from the Cardiovascular Endpoints For Obstructive Sleep Apnea with Twelfth Cranial Nerve Stimulation (CARDIOSA-12) trial: Sleep-related functional outcomes in CARDIOSA-12
Source: J Clin Sleep Med. 2026 Apr 7;22(1):45. doi: 10.1007/s44470-026-00067-x (PMC13057149; doi:10.1007/s44470-026-00067-x)
Supplement: Supplementary file 3 — (DOCX.46.8 KB) [file 44470_2026_67_MOESM3_ESM.docx]

**Online Supplemental Material**

**Cardiovascular Endpoints for Obstructive Sleep Apnea with Twelfth Cranial Nerve Stimulation (CARDIOSA-12): Subjective and Objective Measures of Sleep-related Function**

Akshay Tangutur, MS; Phoebe K. Yu, MD; Mathias Basner, MD, PhD, MSC; Brendan T. Keenan, MS; Raj C. Dedhia, MD, MSCR

**Supplemental Methods**

*Patient Reported Outcomes*

Epworth Sleepiness Scale (ESS). The ESS is an 8-item screening questionnaire that evaluates sleepiness.^1^ Participants were asked to rate their likelihood of dozing off in various situations using a Likert scale, where higher values indicated higher likelihood of dozing off. Scores were interpreted as follows: normal daytime sleepiness (≤10), mild excessive daytime sleepiness (11-12), moderate excessive daytime sleepiness (13-15), and severe excess daytime sleepiness (≥16).

Functional Outcomes of Sleep Questionnaire-10 (FOSQ-10). The FOSQ-10 is a validated, shortened, 10-item adaptation of the original Functional Outcomes of Sleep Questionnaire that evaluates the functional impact of a patient’s daytime sleepiness.^2^ Participants were asked to rate the impact of their daytime sleepiness on various activities with lower values (1) indicating higher effect and higher values (4) indicated lowest effect. The total score was calculated as a mean-weighted item score. For subscales with multiple items (i.e., General Productivity, Activity Level, Vigilance), the mean of non-missing responses was computed, and the total score was calculated by averaging the subscale means and multiplying by five. Items that were left blank or marked as “not applicable” were excluded from subscale means to prevent score distortion.

Insomnia Severity Index (ISI). The ISI is a validated, 7-item screening questionnaire that evaluates severity of insomnia symptoms.^3^ Participants were asked to rate the severity of their insomnia-related symptoms using a Likert scale, where higher scores indicated more severe symptoms. Each item is scored from 0 to 4, and the total score is calculated by summing all the item scores, yielding a possible total score range from 0 to 28.

Snoring Visual Analogue Scale (Snoring VAS). The Snoring VAS is a standardized, objective method of assessing how bothersome snoring is to the participant and/or bed partner. Participants were asked to rate using a visual analogue scale, where higher values indicated snoring as more bothersome.^4^

**Supplemental References**

E1. Johns MW. A new method for measuring daytime sleepiness: the Epworth sleepiness scale. *Sleep*. 1991;14(6):540-545. doi:10.1093/sleep/14.6.540

E2. Chasens ER, Ratcliffe SJ, Weaver TE. Development of the FOSQ-10: a short version of the Functional Outcomes of Sleep Questionnaire. *Sleep*. 2009;32(7):915-919. doi:10.1093/sleep/32.7.915

E3. Morin CM, Belleville G, Bélanger L, Ivers H. The Insomnia Severity Index: psychometric indicators to detect insomnia cases and evaluate treatment response. *Sleep*. 2011;34(5):601-608. doi:10.1093/sleep/34.5.601

E4. Alqurashi YD, Dawidziuk A, Alqarni A, et al. A visual analog scale for the assessment of mild sleepiness in patients with obstructive sleep apnea and healthy participants. *Ann Thorac Med*. 2021;16(2):141-147. doi:10.4103/atm.ATM_437_20

**Table S1.** Demographics of the Per Protocol Analysis Set

| **Characteristic** | **Mean (**±**SD) or %** | | | **p*** |
| --- | --- | --- | --- | --- |
|  | **All** | **Control First** | **Active First** |  |
| Total N | 20 | 12 | 8 | – |
| Gender, % |  |  |  |  |
| Male | 50.0% | 50.0% | 50.0% | >0.999 |
| Female | 50.0% | 50.0% | 50.0% |  |
| Age, years | 71.0±10.2 | 70.3±12.8 | 72.1±4.6 | 0.673 |
| <70 | 45.0% | 41.7% | 50.0% | 0.714 |
| ≥70 | 55.0% | 58.3% | 50.0% |  |
| Race, % |  |  |  |  |
| White | 95.0% | 91.7% | 100.0% | 0.402 |
| Black | 0.0% | 0.0% | 0.0% |  |
| Hispanic | 0.0% | 0.0% | 0.0% |  |
| Asian | 5.0% | 8.3% | 0.0% |  |
| BMI, kg/m^2^ | 27.6±3.6 | 26.9±2.8 | 28.5±4.7 | 0.395 |
| <30 | 65.0% | 83.3% | 37.5% | 0.035 |
| ≥30 | 35.0% | 16.7% | 62.5% |  |
| Diabetes, % | 27.8% | 30.0% | 25.0% | 0.814 |
| Hypertension, % | 55.6% | 50.0% | 62.5% | 0.560 |
| Hypertension Meds, % | 55.6% | 50.0% | 62.5% | 0.560 |
| Smoking Status, % |  |  |  |  |
| Current | 10.0% | 8.3% | 12.5% | 0.053 |
| Never | 45.0% | 66.7% | 12.5% |  |
| Former | 45.0% | 25.0% | 75.0% |  |
| Baseline AHI, events/hour | 31.6±12.7 | 31.9±11.3 | 31.2±15.4 | 0.912 |
| HGNS Configuration |  |  |  |  |
| +/-/+ | 90.0% | 91.7% | 87.5% | 0.761 |
| -/-/- | 0.0% | 0.0% | 0.0% |  |
| -/○/- | 0.0% | 0.0% | 0.0% |  |
| ○/-/○ | 10.0% | 8.3% | 12.5% |  |
| ^*^p-value from T-test or chi-squared test comparing participants randomized to control condition first or active therapy first | | | | |

**Table S2.** Demographics of the modified Intent-to-Treat Analysis Set for Cognitive Testing

| **Characteristic** | **Mean (**±**SD) or %** | | | **p*** |
| --- | --- | --- | --- | --- |
|  | **All** | **Control First** | **Active First** |  |
| Total N | 43 | 22 | 21 | – |
| Gender, % |  |  |  |  |
| Male | 69.8% | 68.2% | 71.4% | 0.817 |
| Female | 30.2% | 31.8% | 28.6% |  |
| Age, years | 65.7±10.2 | 63.7±11.8 | 67.8±7.83 | 0.182 |
| <70 | 62.8% | 68.2% | 57.1% | 0.454 |
| ≥70 | 37.2% | 31.8% | 42.9% |  |
| Race, % |  |  |  |  |
| White | 93.0% | 90.9% | 95.2% | 0.395 |
| Black | 2.3% | 0.0% | 4.8% |  |
| Hispanic | 2.3% | 4.6% | 0.0% |  |
| Asian | 2.3% | 4.6% | 0.0% |  |
| BMI, kg/m^2^ | 29.7±4.6 | 29.3±3.9 | 30.1±5.3 | 0.580 |
| <30 | 51.2% | 59.1% | 42.9% | 0.287 |
| ≥30 | 48.8% | 40.9% | 57.1% |  |
| Diabetes, % | 27.9% | 18.2% | 38.1% | 0.146 |
| Hypertension, % | 55.8% | 54.6% | 57.1% | 0.864 |
| Hypertension Meds, % | 58.1% | 54.6% | 61.9% | 0.625 |
| Smoking Status, % |  |  |  |  |
| Current | 6.98% | 13.6% | 0.0% | 0.061 |
| Never | 46.5% | 54.6% | 38.1% |  |
| Former | 46.5% | 31.8% | 61.9% |  |
| Baseline AHI, events/hour | 34.2±14.9 | 32.7±16.1 | 35.8±14.0 | 0.503 |
| HGNS Configuration |  |  |  |  |
| +/-/+ | 72.1% | 68.2% | 76.2% | 0.304 |
| -/-/- | 4.65% | 0.0% | 9.52% |  |
| -/○/- | 6.98% | 9.1% | 4.76% |  |
| ○/-/○ | 16.3% | 22.7% | 9.52% |  |
| ^*^p-value from T-test or chi-squared test comparing participants randomized to control condition first or active therapy first | | | | |

**Table S3.** Demographics of the Per Protocol Analysis Set for Cognitive Testing

| **Characteristic** | **Mean (**±**SD) or %** | | | **p*** |
| --- | --- | --- | --- | --- |
|  | **All** | **Control First** | **Active First** |  |
| Total N | 15 | 9 | 6 | – |
| Gender, % |  |  |  |  |
| Male | 46.7% | 44.4% | 50.0% | 0.833 |
| Female | 53.3% | 55.6% | 50.0% |  |
| Age, years | 69.6±10.7 | 67.1±13.0 | 73.3±4.71 | 0.224 |
| <70 | 46.7% | 55.6% | 33.3% | 0.398 |
| ≥70 | 53.3% | 44.4% | 66.7% |  |
| Race, % |  |  |  |  |
| White | 93.3% | 88.9% | 100% | 0.398 |
| Asian | 6.7% | 11.1% | 0.0% |  |
| BMI, kg/m^2^ | 28.1±3.7 | 27.3±3.1 | 29.4±4.4 | 0.323 |
| <30 | 60.0% | 77.8% | 33.3% | 0.085 |
| ≥30 | 40.0% | 22.2% | 66.7% |  |
| Diabetes, % | 33.3% | 33.3% | 33.3% | >0.999 |
| Hypertension, % | 60.0% | 44.4% | 83.3% | 0.132 |
| Hypertension Meds, % | 60.0% | 44.4% | 83.3% | 0.132 |
| Smoking Status, % |  |  |  |  |
| Current | 6.7% | 11.1% | 0.0% | 0.065 |
| Never | 46.7% | 66.7% | 16.7% |  |
| Former | 46.7% | 22.2% | 83.3% |  |
| Baseline AHI, events/hour | 31.0±13.2 | 28.4±11.0 | 34.9±16.3 | 0.420 |
| HGNS Configuration |  |  |  |  |
| +/-/+ | 86.7% | 88.9% | 83.3% | 0.757 |
| ○/-/○ | 13.3% | 11.1% | 16.7% |  |
| ^*^p-value from T-test or chi-squared test comparing participants randomized to control condition first or active therapy first | | | | |

**Table S4.** Comparisons of outcomes in mITT analysis set in patient subgroups

| **Subgroup Variable** | ***Difference (95% CI) between Within-Strata Estimates*** | **N** | **Mean (95% CI) Difference**^†^ | **N** | **Mean (95% CI) Difference**^†^ |
| --- | --- | --- | --- | --- | --- |
| ***Treatment Order*** | | ***Control First*** | | ***Active First*** | |
| Cognitive Measures |  |  |  |  |  |
| PVT (Mean RRT)^§^ | -0.07 (-0.23, 0.10) | 22 | 0.08 (-0.05, 0.22) | 21 | 0.01 (-0.09, 0.11) |
| **PVT (Lapses)^‡^** | **2.81 (0.92, 4.71)** | **22** | **-1.91 (-3.56, -0.26)** | **21** | **0.90 (-0.09, 1.90)** |
| PVT (False Starts)^‡^ | -0.23 (-1.15, 0.69) | 22 | 0.55 (-0.12, 1.21) | 21 | 0.32 (-0.36, 1.00) |
| **DSST (Mean RT)^‡^** | **129.1 (1.4, 256.8)** | **22** | **-110.0 (-209.3, -10.7)** | **21** | **19.1 (-66.6, 104.8)** |
| DSST (# Correct)^§^ | 0.00 (-0.03, 0.04) | 22 | -0.01 (-0.04, 0.02) | 21 | -0.01 (-0.02, 0.01) |
| **DSST (% Accuracy)^§^** | **-3.52 (-6.59, -0.45)** | **22** | **2.00 (-0.56, 4.56)** | **21** | **-1.52 (-3.33, 0.28)** |
| ESS Total^‡^ | 0.13 (-1.63, 1.89) | 31 | -1.81 (-2.88, -0.73) | 28 | -1.68 (-3.15, -0.21) |
| Snoring VAS^‡^ | -5.24 (-21.44, 10.96) | 30 | -13.07 (-22.20, -3.93) | 26 | -18.31 (-32.70, -3.91) |
| ISI Total^‡^ | -1.47 (-4.24, 1.31) | 31 | -1.68 (-3.43, 0.08) | 28 | -3.14 (-5.41, -0.87) |
| FOSQ Total^§^ | -0.07 (-1.03, 0.88) | 31 | 0.91 (0.25, 1.58) | 28 | 0.84 (0.12, 1.56) |
| ***PVT Mean Reciprocal Response Time*** | | ***Below Median*** | | ***Above Median*** | |
| Cognitive Measures |  |  |  |  |  |
| **PVT (Mean RRT)^§^** | **-0.18 (-0.34, -0.02)** | **20** | **0.15 (0.02, 0.27)** | **23** | **-0.04 (-0.14, 0.07)** |
| **PVT (Lapses)^‡^** | **2.08 (0.09, 4.08)** | **20** | **-1.65 (-3.54, 0.24)** | **23** | **0.43 (-0.56, 1.43)** |
| PVT (False Starts)^‡^ | -0.36 (-1.28, 0.56) | 20 | 0.63 (0.08, 1.18) | 23 | 0.27 (-0.47, 1.01) |
| DSST (Mean RT)^‡^ | 92.7 (-38.4, 223.8) | 20 | -96.5 (-199.5, 6.5) | 23 | -3.8 (-92.7, 85.0) |
| DSST (# Correct)^§^ | 0.03 (0.00, 0.07) | 20 | -0.03 (-0.06, 0.01) | 23 | 0.01 (-0.01, 0.02) |
| DSST (% Accuracy)^§^ | 0.52 (-2.75, 3.79) | 20 | 0.00 (-2.58, 2.58) | 23 | 0.52 (-1.68, 2.72) |
| ESS Total^‡^ | 0.97 (-1.27, 3.22) | 20 | -2.80 (-4.31, -1.29) | 23 | -1.83 (-3.54, -0.11) |
| **Snoring VAS^‡^** | **16.65 (1.75, 31.55)** | **20** | **-24.65 (-38.87, -10.43)** | **23** | **-8.00 (-15.33, -0.67)** |
| ISI Total^‡^ | 1.11 (-1.64, 3.86) | 20 | -3.50 (-5.42, -1.58) | 23 | -2.39 (-4.44, -0.34) |
| FOSQ Total^§^ | 0.06 (-1.09, 1.20) | 20 | 1.05 (0.05, 2.06) | 23 | 1.11 (0.44, 1.78) |
| ***DSST Mean Response Time*** | | ***Below Median*** | | ***Above Median*** | |
| Cognitive Measures |  |  |  |  |  |
| PVT (Mean RRT)^§^ | -0.04 (-0.21, 0.13) | 21 | 0.07 (-0.08, 0.22) | 22 | 0.03 (-0.06, 0.12) |
| PVT (Lapses)^‡^ | -0.58 (-2.66, 1.50) | 21 | -0.24 (-2.01, 1.53) | 22 | -0.82 (-2.07, 0.43) |
| PVT (False Starts)^‡^ | 0.48 (-0.43, 1.39) | 21 | 0.19 (-0.19, 0.57) | 22 | 0.67 (-0.17, 1.51) |
| **DSST (Mean RT)^‡^** | **-131.7 (-259.2, -4.3)** | **21** | **20.5 (-42.9, 83.8)** | **22** | **-111.3 (-224.4, 1.9)** |
| DSST (# Correct)^§^ | 0.01 (-0.02, 0.05) | 21 | -0.02 (-0.05, 0.02) | 22 | 0.00 (-0.02, 0.02) |
| DSST (% Accuracy)^§^ | 2.41 (-0.77, 5.58) | 21 | -0.95 (-3.49, 1.59) | 22 | 1.45 (-0.63, 3.54) |
| ESS Total^‡^ | 2.06 (-0.11, 4.23) | 21 | -3.33 (-5.16, -1.51) | 22 | -1.27 (-2.59, 0.05) |
| Snoring VAS^‡^ | 6.55 (-9.07, 22.17) | 21 | -19.10 (-31.54, -6.65) | 22 | -12.55 (-22.88, -2.21) |
| ISI Total^‡^ | 1.02 (-1.73, 3.77) | 21 | -3.43 (-5.32, -1.53) | 22 | -2.41 (-4.51, -0.31) |
| **FOSQ Total^§^** | **-1.62 (-2.64, -0.60)** | **21** | **1.91 (1.07, 2.75)** | **22** | **0.29 (-0.35, 0.94)** |
| **Bold** indicates measures with differences in change between subgroups (interaction p<0.05); ^†^Difference calculated as active therapy minus control condition; ‡Decreases with active therapy considered positive treatment benefit; §Increases with active therapy considered positive treatment benefit; | | | | | |

**Supplemental Figure Legends**

Figure S1. Comparison of Psychomotor Vigilance (PVT) Outcomes in Per Protocol (PP) Analysis Set. PVT indicates Psychomotor Vigilance Test; RRT indicates Reciprocal Reaction Time.

Figure S2. Comparison of Digit Symbol Substitution Task (DSST) Outcomes in Per Protocol (PP) Analysis Set. DSST indicates Digit Symbol Substitution Test; RT indicates Reaction Time.
